# Supplementary material for: Seroepidemiology of Measles, Mumps and Rubella on Bonaire, St. Eustatius and Saba: The First Population-Based Serosurveillance Study in Caribbean Netherlands
Source: Vaccines (Basel). 2019 Oct 1;7(4):137. doi: 10.3390/vaccines7040137 (PMC6963433; doi:10.3390/vaccines7040137)
Supplement: Supplementary file 1 [file vaccines-07-00137-s001.zip › Supplement Figures (vaccines-583487)/RA Vos et al (vaccines-583487)-Figure S3ABC.pdf]

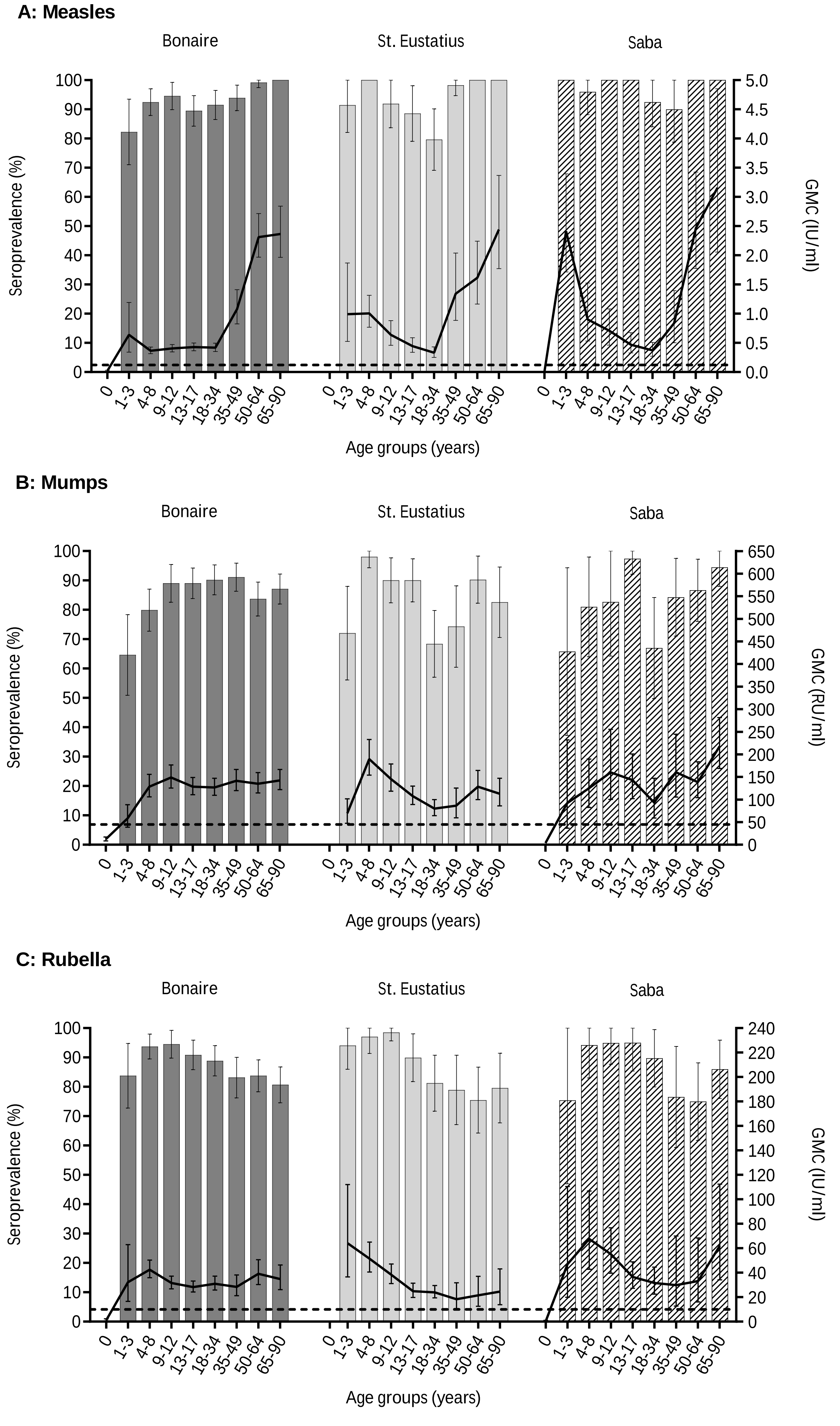

Figure S3. Age-specific seroprevalence (%) and geometric mean concentration (GMC) (with 95% confidence intervals) of measles (A), mumps (B), and rubella (C) IgG antibodies in the general population of Bonaire, St. Eustatius and Saba, 2017. Note: antibody concentrations ≥0.120 international units (IU)/ml for measles, ≥45.0 RIVM units (RU)/ml for mumps, and ≥10.0 IU/ml for rubella were considered seropositive (dashed lines).
