# Supplementary material for: Seroepidemiology of Measles, Mumps and Rubella on Bonaire, St. Eustatius and Saba: The First Population-Based Serosurveillance Study in Caribbean Netherlands
Source: Vaccines (Basel). 2019 Oct 1;7(4):137. doi: 10.3390/vaccines7040137 (PMC6963433; doi:10.3390/vaccines7040137)
Supplement: Supplementary file 1 [file vaccines-07-00137-s001.zip › Supplement Figures (vaccines-583487)/RA Vos et al (vaccines-583487)-Figure S4ABCDEF.pdf]

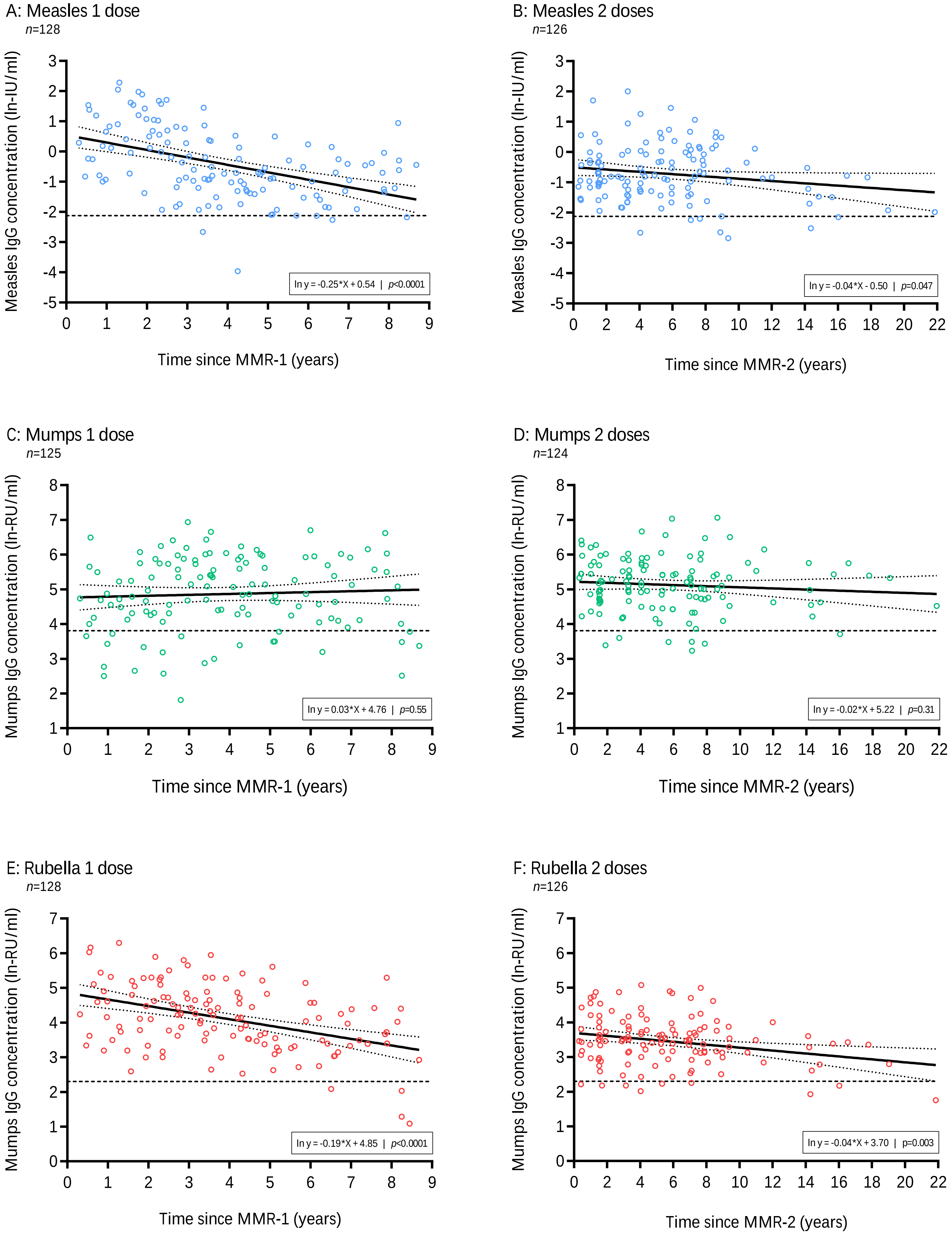

Figure S4. Persistence of measles (A & B), mumps (C & D), and rubella (E & F) IgG antibodies (ln-international units (IU) or RIVM units (RU)/ml)) after one and two doses of MMR-vaccination among participants from the Dutch overseas territories. Note: the solid line represents the fitted model via linear regression analyses, the small dotted lines the 95% confidence intervals, and the dashed lines the ln-cutoff for seropositivity.
