# Supplementary figures and images for: Seroepidemiology of Measles, Mumps and Rubella on Bonaire, St. Eustatius and Saba: The First Population-Based Serosurveillance Study in Caribbean Netherlands
Source: Vaccines (Basel). 2019 Oct 1;7(4):137. doi: 10.3390/vaccines7040137 (PMC6963433; doi:10.3390/vaccines7040137)

**Figure S2.** Flowchart of the study

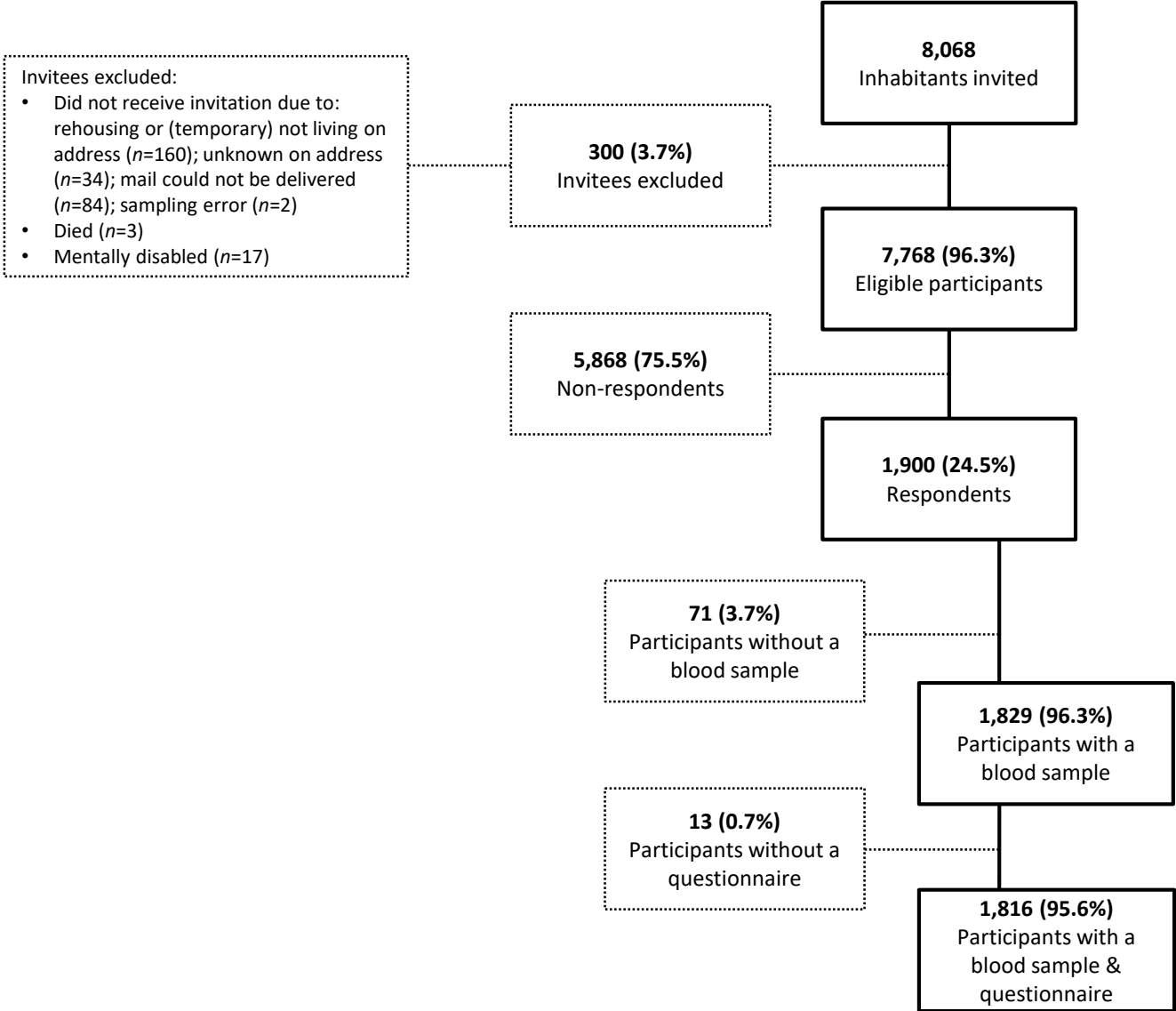

Supplement: Supplementary file 1 [file vaccines-07-00137-s001.zip › Supplement Figures (vaccines-583487)/RA Vos et al (vaccines-583487)-Figure S2.pdf]
